# Inter-Species Complementation of the Translocon Beta Subunit Requires Only Its Transmembrane Domain

**DOI:** 10.1371/journal.pone.0003880

**Published:** 2008-12-05

**Authors:** Alexandre Leroux, Luis A. Rokeach

**Affiliations:** Department of Biochemistry, Université de Montréal, Montréal, Québec, Canada; Wellcome Trust Sanger Institute, United Kingdom

## Abstract

In eukaryotes, proteins enter the secretory pathway through the translocon pore of the endoplasmic reticulum. This protein translocation channel is composed of three major subunits, called Sec61α, β and γ in mammals. Unlike the other subunits, the β subunit is dispensable for translocation and cell viability in all organisms studied. Intriguingly, the knockout of the Sec61β encoding genes results in different phenotypes in different species. Nevertheless, the β subunit shows a high level of sequence homology across species, suggesting the conservation of a biological function that remains ill-defined. To address its cellular roles, we characterized the homolog of Sec61β in the fission yeast *Schizosaccharomyces pombe* (Sbh1p). Here, we show that the knockout of *sbh1* ^+^ results in severe cold sensitivity, increased sensitivity to cell-wall stress, and reduced protein secretion at 23°C. Sec61β homologs from *Saccharomyces cerevisiae* and human complement the knockout of *sbh1* ^+^ in *S. pombe*. As in *S. cerevisiae*, the transmembrane domain (TMD) of *S. pombe* Sec61β is sufficient to complement the phenotypes resulting from the knockout of the entire encoding gene. Remarkably, the TMD of Sec61β from *S. cerevisiae* and human also complement the gene knockouts in both yeasts. Together, these observations indicate that the TMD of Sec61β exerts a cellular function that is conserved across species.

## Introduction

Protein secretion is an essential process in all organisms. In eukaryotes, entry of proteins into the secretory pathway requires passage across the endoplasmic reticulum (ER) lipid bilayer. Translocation of proteins into the ER is accomplished through a protein conduction channel (PCC) [1], [2]. The PCC is composed of a highly conserved heterotrimeric core called the Sec61αβγ complex in eukaryotes, and the SecYEG complex in bacteria [3], [4], [5]. The PCC associates with several soluble proteins to form the translocon, the ER translocation machinery [6], [7]. The translocon functions in co-translational translocation of nascent proteins, as well as in the retro-translocation of misfolded polypeptides for degradation in the cytosol by the ER-associated degradation (ERAD) mechanism [3], [8], [9], [10], [11]. In addition, the association of the translocon with the tetrameric Sec62/63 complex and the BiP molecular chaperone forms the Sec complex, which is involved in post-translational translocation [12], [13], [14]. The genomes of all organisms sequenced to date encode at least one homolog of each Sec61αβγ/SecYEG subunits, suggesting that these proteins have a universal cellular role [15].

So far, the functions of the α and γ subunits of the PCC are the best understood. The α subunit consists of ten transmembrane segments assembled in an hourglass shape, forming the pore of the channel [16], [17] and acting as the main ribosome receptor [18], [19]. The γ subunit is a single-span membrane protein that binds and stabilizes the translocon pore by linking the two halves of the α subunit [16], [20]. By contrast, the precise function of the β subunit, a 10 kD C-tail-anchored transmembrane protein, remains elusive. Interestingly, Sec61β and its prokaryotic counterpart SecG are the only translocon subunits that are not essential for cell viability in yeast and bacteria [21], [22], [23], [24]. Moreover, mammalian Sec61β is not required for protein translocation *in vitro*, but has a kinetic effect on co-translational translocation [25]. Likewise, bacterial SecG is dispensable for translocation in reconstituted membrane vesicles, but has a stimulatory effect in this process [26]. In *S. cerevisiae*, the double knockout of the genes encoding the two paralogs of the translocon β subunits (*SBH1* and *SBH2*) has only a moderate effect on translocation of reporter proteins [22], [24], [27], [28]. Regarding protein secretion, the relevance of the function of Sec61β in co- and post-translational translocation remains unclear as the knockout of the translocon β subunits reduces α-amylase secretion in *S. cerevisiae*, but has no effect in *Yarrowia lipolytica* [23], [27].

Based on the phenotypes resulting from its knockout, several cellular roles for the translocon β subunit have been proposed. In prokaryotes, SecG facilitates the membrane insertion/de-insertion cycle of the SecA ATPase, the bacterial translocation motor [29], [30]. In *S. cerevisiae*, Sbh1p was proposed to act as the guanine nucleotide exchange factor for the signal recognition particle receptor (SR) [31], whereas Sbh2p was suggested to mediate recognition of vacant translocons by functional interactions with the SR [28]. Sbh1p also rescues several exocyst mutants in yeasts and coimmunoprecipitates with Sec10p, Sec6p and Sec8p in mammalian cells, three components of the exocyst complex, suggesting a role in vesicular transport [24], [32], [33]. The translocon β subunit was also shown to be recruited to the ER-derived quality control compartment in ERAD [34], and to have an accessory function in the unfolded protein response in different organisms [23], [35], [36], [37]. The diversity of functions proposed for the translocon β subunit suggests that different homologs of this protein have species-specific roles.

Recently, Feng *et al.* [27] demonstrated that the transmembrane domain (TMD) of *S. cerevisiae* Sbh1p or Sbh2p are necessary and sufficient to suppress the phenotypes of *sbh1*Δ*sbh2*Δ cells, including heat sensitivity at 38°C, and co-translational translocation defects. This was further confirmed in another study, where N-terminal truncation mutants of Sbh2p restored normal integration of DPAPB and translocation of CPY in the *sbh1*Δ*sbh2*Δ strain [28]. Furthermore, the TMD of Sbh1p is sufficient for co-immunoprecipitation with the translocon α and γ subunits, indicating correct integration and interaction at the Sec61 translocon site [27]. The TMD of Sbh1p also interacts with Rtn1p, a protein involved in structuring the cortical ER, in absence of other translocon subunits, suggesting a role for the β subunit outside the translocation apparatus [27]. Taken together, these results suggest that the TMD is the critical region responsible for the undefined function of Sec61β.

Here, we analyzed the functional conservation of the translocon β subunit and its TMD. We show that the translocon β subunit is not essential in fission yeast at 30°C. However, it is required for growth at low temperature, and its deletion causes SDS sensitivity and reduced protein secretion at 23°C. Overexpression of *sbh1* ^+^ also diminishes protein secretion of a model protein, increases sensitivity to cell-wall stress, and causes morphological abnormalities. The phenotypes associated with *sbh1* ^+^ deletion are suppressed by the 26 amino acids of the TMD. Remarkably, the TMDs from both *S. cerevisiae* homologs of the translocon β subunit and that from human also complement the deletion phenotypes in fission and budding yeast. These results demonstrate that the TMD function of Sec61β is conserved and sufficient to exert most biological roles of the full-length protein. These observations pose Sec61β as a model to study alternative functions of TMDs apart from the anchoring to the ER membrane.

## Results

### *S. pombe* encodes a single Sec61β homolog

**Figure 1:**
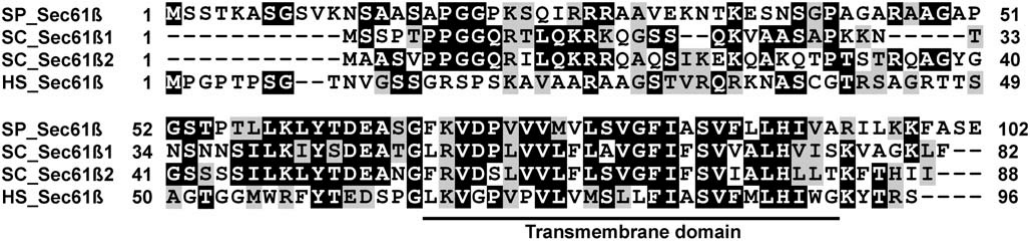
The translocon β subunit is conserved from yeast to human. Amino-acid sequence comparison between translocon beta subunits of *S. pombe* (SP_Sec61β), *S. cerevisiae* (SC_Sec61β1 and SC_Sec61β2) and human (HS_Sec61β). Identical amino acids are shaded in black, similar amino acids are shaded in grey. The predicted conserved transmembrane domain is underlined.

Based on Wu-Blast2 analyses of the fission yeast proteome [38], *S. pombe* encodes a single Sec61β homolog at ORF SPBC2G2.03c (*sbh1* ^+^), hereafter designed as SP_Sec61β for the sake of clarity. The deduced primary sequence of the protein is presented in Figure 1. As expected for a translocon subunit, the predicted protein displays a high level of sequence conservation with both paralogs of *S. cerevisiae* (SC) and that of human (HS). SP_Sec61β is 35% identical (66% similar) to SC_Sec61β1 (Sbh1p), 43% identical (65% similar) to SC_Sec61β2 (Sbh2p), and 29% identical (58% similar) to HS_Sec61β (human Sec61β). Multiple sequence alignment using Clustal W2 [39] revealed that SP_Sec61β is evolutionary closer to HS_Sec61β than its *S. cerevisiae* counterparts (not shown), thus validating our choice of *S. pombe* as a model organism to study the translocon β subunit of higher eukaryotes. Analyses of SP_Sec61β using various ExPASy proteomic tools [40] allowed the identification of a single domain, constituted of a continuous stretch of 26 amino acids near the C-terminus. This hydrophobic tail is predicted to form an alpha helix crossing the lipid bilayer once, with the N-terminus of the protein exposed to the cytoplasm. Thus, as in *S. cerevisiae* and human, SP_Sec61β is predicted to be a tail-anchored protein. Interestingly, the TMDs of Sec61β homologs are much more conserved than their N-terminal domains, 53–61% identical from yeast to human as compared to only 18–41% for the cytosolic part. In comparison, the tail anchor of the translocon γ subunit is only 40–50% identical from yeast to human, a level of sequence homology comparable to the entire protein, which is 44–48% identical. This conservation suggests a critical function for the TMD region of Sec61β homologs, not solely explained by its role as a membrane anchor, especially as almost any stretch of hydrophobic amino acids can perform this function [41].

### Overexpression of SP_Sec61β induces morphological changes and cell-wall sensitivity

**Figure 2:**
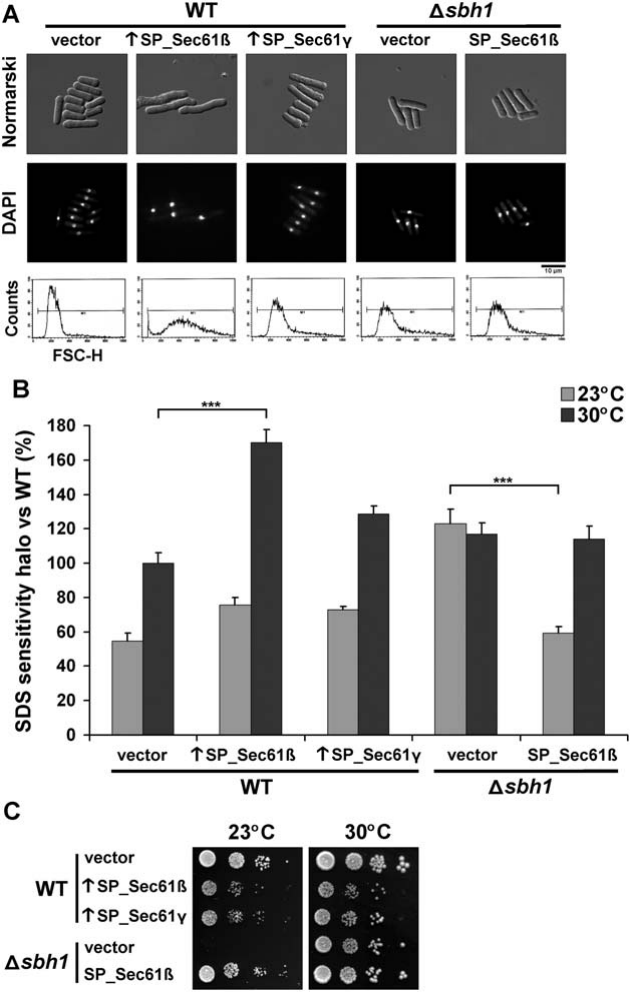
Phenotypes associated with the knockout and overexpression of SP_Sec61β. (A) Morphologic comparison between strains. WT cells (SP556), cells overexpressing *S. pombe* translocon β subunit (↑SP_Sec61β) or γ subunit (↑SP_Sec61γ) and cells deleted for the SP_Sec61β encoding gene (SP15039, Δ*sbh1*) with or without a plasmid encoding SP_Sec61β were cultured to exponential phase, stained with DAPI (as nuclear marker) and analyzed by Normarski (upper panel) or fluorescent microscopy (middle panel). Cell size distribution was assessed by cell sorting of 10,000 cells (lower panel). (B) SDS sensitivity comparison between strains. Three exponential cultures of WT and Δ*sbh1* cells bearing the indicated plasmids were spread on top-agar MM+AL, following which a drop of 10 µL of SDS 10% was added on the center of each plate. After 3–5 days of incubation at the indicated temperatures, the area of the dead-cell halo was measured and reported to the WT strain at 30°C (100%). Data shown are mean±standard deviation of three or more independent experiments. *** indicates *p*<0.001 for Student's t-test. (C) Growth of WT and Δ*sbh1* strains devoid or overexpressing SP_Sec61β or SP_Sec61γ at different temperatures. Exponential cultures were serially-diluted (10^−1^–10^−4^) and spotted on EMM+AL plates. Growth was monitored for 7 days at the indicated temperatures. Results are representative of three or more independent experiments.

To explore its cellular roles in *S. pombe*, we overexpressed the translocon β subunit expecting to exacerbate its function and induce a revealing phenotype. The ORF of SP_Sec61β was cloned under the control of the strong thiamine-repressible *nmt* promoter in a pREP2 vector [42]. As shown in Figure 2A (upper panel), overexpression of SP_Sec61β resulted in a elongated phenotype with a single nucleus per cell (see DAPI staining, middle panel). This phenotype was specific to the translocon β subunit because the overexpression of *S. pombe* γ subunit (SP_Sec61γ) had no effect on cell morphology. Moreover, the elongated phenotype was reverted by addition of thiamine, which represses the promoter; and the phenotype was not observed when expression was driven by a weaker nmt* promoter (not shown). The elongated phenotype caused by overproduction of SP_Sec61β was further confirmed by global population analysis using flow cytometry (FACS). As shown in Figure 2A (lower panel), cells overexpressing SP_Sec61β had an average size (FSC-H) value 1.9 higher than WT. The wider size distribution can be explained by the presence of cells with various altered morphologies, which were either more granulated, had bigger vesicles or displayed an aberrant number of septa in ∼10% of cases (not shown).

To assess if the elongated phenotype is associated with membrane and/or cell-wall defects, we performed an SDS resistance assay at various temperatures (Figure 2B). SDS is a strong detergent that disturbs the cytoplasmic membrane when the cell-wall is compromised, thus resulting in a dead-cell halo that can be measured [43]. In these conditions, cells overexpressing SP_Sec61β were up to 1.8 more sensitive to SDS as compared to the vector control at 30°C. In comparison, cells overexpressing SP_Sec61γ were only 1.3 times more sensitive at this temperature. At 23°C, the strains overproducing the β or γ subunits were both 1.4 times more sensitive than the vector control. As addition of 1.2 M sorbitol, an osmotic stabilizer [44], did not rescue the SDS sensitivity nor the elongated cell size (not shown), it is unlikely that these phenotypes are only due to an incorrect cell-wall formation. These results demonstrate that the overexpression of SP_Sec61β alters cellular morphology, accompanied by a higher sensitivity to membrane stress, suggesting a potential role of this protein in the coordination between cell-membrane growth and the cell-cycle in fission yeast.

### Cells depleted of SP_Sec61β are cold-sensitive

To investigate whether SP_Sec61β is essential for viability, a *sbh1* ^+^/Δ*sbh1::kan* ^R^ diploid strain was sporulated to analyze its meiotic progeny. All spores were viable on YE medium, with a 2:2 segregation of the *kan* ^R^ marker. Deletion of *sbh1* ^+^ was confirmed by Northern blotting, Southern blotting and PCR, demonstrating that SP_Sec61β is not essential for viability (not shown). In contrast to the overexpression of SP_Sec61β, the deletion of *sbh1* ^+^ did not induce morphological changes (Figure 2A). As it was shown in *S. cerevisiae* that deletion of the genes encoding the two translocon β subunits (*SBH1* and *SBH2*) results in heat sensitivity at 38°C [22], [24], we measured the growth of *S. pombe* Δ*sbh1* cells at different temperatures. In contrast to *S. cerevisiae*, Δ*sbh1* cells from *S. pombe* were not heat-sensitive (not shown), but the deletion resulted in severe cold sensitivity at 23°C (Figure 2C). Normal growth was not restored by replacing the cells at 30°C after incubation at 23°C for 7 days, indicating that growth is permanently arrested or that most Δ*sbh1* cells are dead (not shown). In addition, Δ*sbh1* cells were 2.2 times more sensitive to SDS than WT cells at 23°C, while sensitivity was similar at 30°C (Figure 2B). Transformation of *sbh1* ^+^ on a plasmid under the medium strength nmt* promoter was sufficient to rescue to WT levels the cold- and SDS-sensitivity (Figure 2B–C). Globally, these results demonstrate that while SP_Sec61β is not essential for cell viability at 30°C, it is critical at low temperature.

### The levels of SP_Sec61β modulate secretion

**Figure 3:**
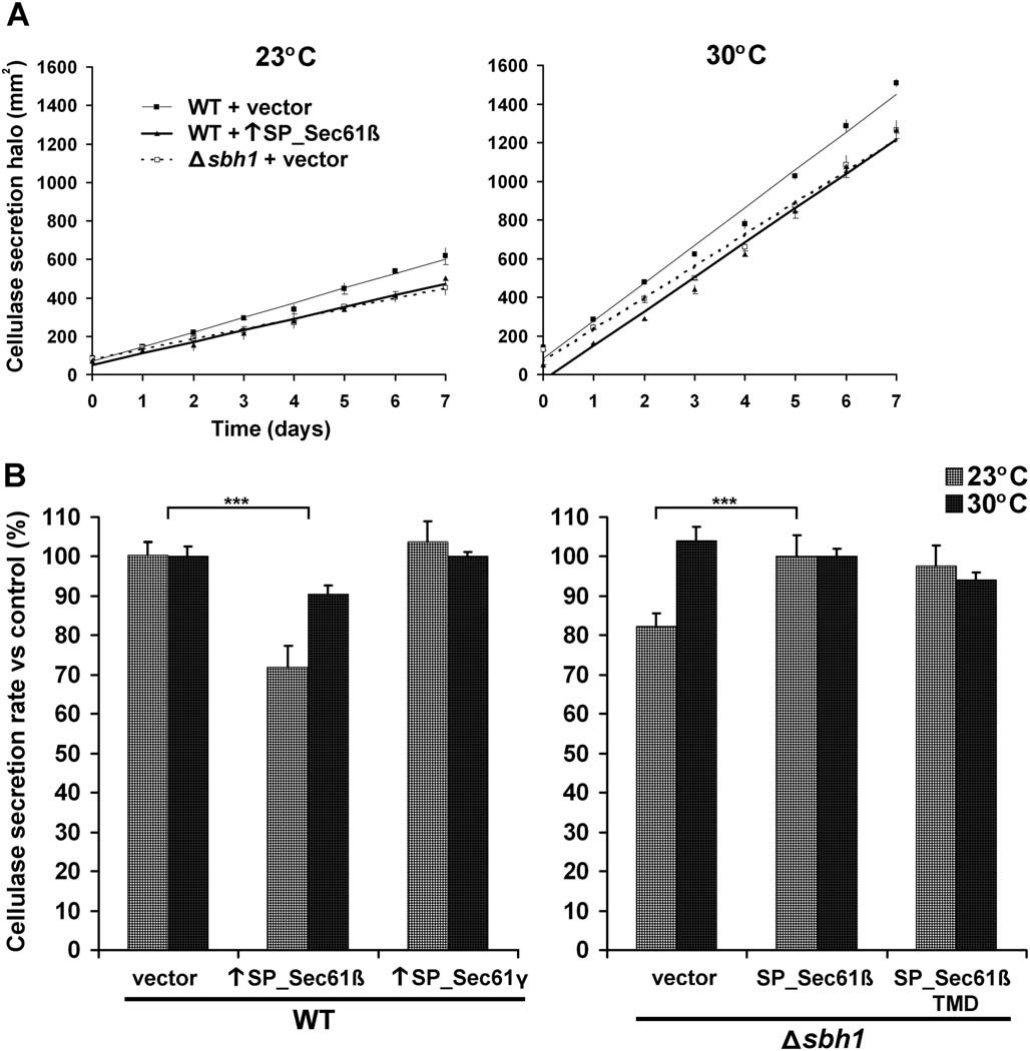
Effects of SP_Sec61β levels on protein secretion. (A) Cellulase secretion efficiency at different temperatures. WT (SP15073) and Δ*sbh1* (SP15074) cells expressing *A. aculeatus* cellulase from a genomic cassette under the control of the *adh1p* promoter, and bearing the empty vector or a plasmid overexpressing (↑) SP_Sec61β were analyzed for secretion. Cells were grown to exponential phase and spotted on EMM+ALH plates supplemented with 0.1% AZCL-HE-cellulose as substrate. The area of the blue halo created after cleavage of the chromogenic substrate by secreted cellulase was monitored for 7 days at 30°C or at 23°C. Time zero represents the moment when area of the halo exceeds the area under the colony. Each point is the mean±standard deviation of three independent cultures. (B) Rate of cellulase secretion at different temperatures. Secretion rates for strains presented in (A) were calculated as the mean slope of three independent cultures, using the WT value (left panel) or the complemented knockout (right panel) at the corresponding temperature as 100% (± standard deviation). WT cells overexpressing SP_Sec61γ and Δ*sbh1* cells bearing a plasmid encoding SP_Sec61β or its transmembrane domain only (TMD) are shown as controls. *** indicates *p*<0.001 for Student's t-test.

Because the knockout of Sec61β was reported to lower protein secretion in *S. cerevisiae*, but not in *Y. lipolytica* [23], [27], we wanted to assess the importance of SP_Sec61β in protein secretion. To this end, we used a previously characterized genomic cassette expressing the reporter protein cellulase from *Aspergillus aculeatus* [45]. The activity of secreted cellulase was measured during 7 days at different temperatures to evaluate the rate of secretion of each strain. As shown in Figure 3B, the secretion rate was not reduced in Δ*sbh1* cells at 30°C, but was reduced to 82% of the control strain at 23°C. Thus, the kinetic effect of the translocon β subunit on secretion is only observed in conditions where secretion is already considerably diminished, such as at low temperature (secretion rate 2.6-fold lower than at 30°C, Figure 3A).

In *S. cerevisiae*, overexpression of the endogenous translocon β subunit or the *Kluyveromyces lactis* homolog increases the secretion efficiency of α-amylase [46]. By contrast, in fission yeast, we show that overproduction of the translocon β subunit reduces the secretion rate of cellulase (Figure 3B). Again, this effect was more pronounced at 23°C (72% of the WT value) than at 30°C (91% of the WT value). This reduction is not an unspecific effect caused by saturation of the ER, as overexpression of the translocon γ subunit did not alter the secretion rate. These results support the idea that even though SP_Sec61β is not essential for growth at normal temperature, a correct SP_Sec61β level is important for efficient protein secretion, especially at low temperature.

### Sec61β is functionally conserved between species

**Figure 4:**
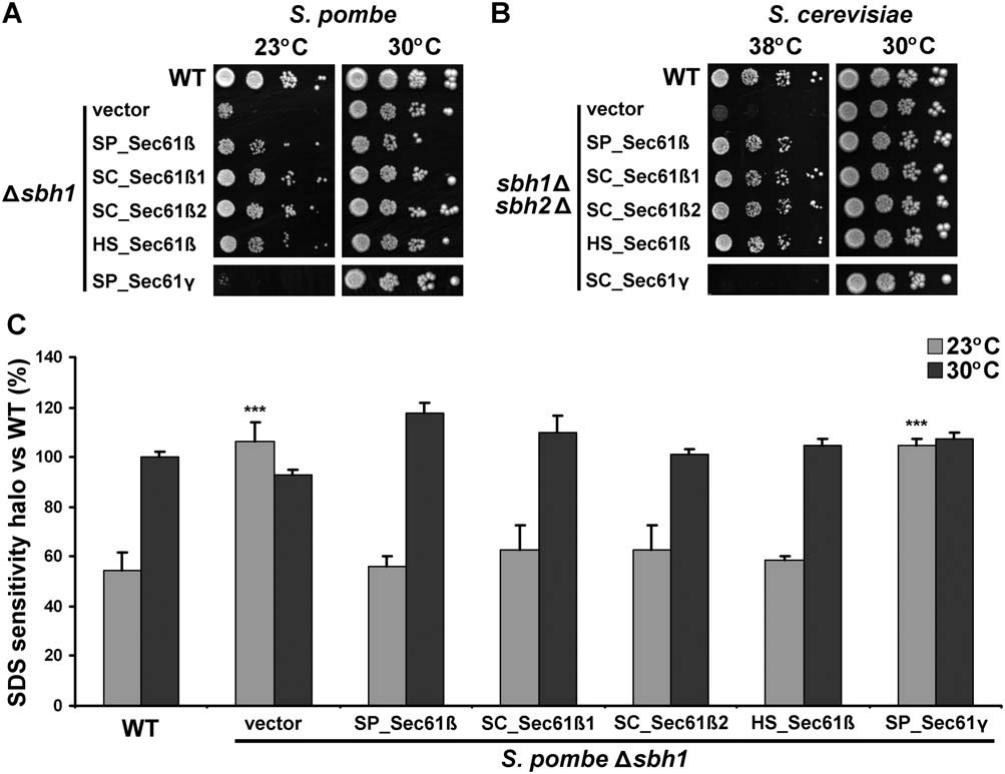
Different homologs complement the phenotypes of the Sec61β knockout in fission and budding yeast. (A) Cold sensitivity of *S. pombe* Δ*sbh1* cells is rescued by different Sec61β homologs. *S. pombe* Δ*sbh1* cells (SP15039) expressing different Sec61β homologs from *S. pombe* (SP), *S. cerevisiae* (SC) or human (HS) were serially-diluted (10^−1^–10^−4^) and spotted on MM+AL plates. Growth was monitored during 7 days at the indicated temperatures. SP_Sec61γ was used as a negative control. Results are representative of three or more independent experiments. (B) Heat sensitivity of *S. cerevisiae sbh1*Δ*sbh2*Δ cells is rescued by different Sec61β homologs. *S. cerevisiae sbh1*Δ*sbh2*Δ cells (SC3232) expressing Sec61β homologs from *S. pombe* (SP), *S. cerevisiae* (SC) or human (HS) were serially-diluted (10^−1^–10^−4^) and spotted on SD-L plates. Growth was monitored during 5 days at the indicated temperatures. SC_Sec61γ was used as a negative control. Results are representative of three or more independent experiments. (C) Suppression of *S. pombe* Δ*sbh1* SDS sensitivity by different Sec61β homologs. SDS-sensitivity halo was measured after 3–5 days of incubation at the indicated temperatures for cultures of the strains presented in (A). Data shown are mean±standard deviation of three or more independent experiments. *** indicates *p*<0.001 for Student's t-test versus WT.

As described above, the knockout of *sbh1* ^+^ results in cold-sensitive phenotypes in *S. pombe*. Interestingly, in budding yeast, the double deletion of both paralogs of Sec61β, hereafter designated as SC_Sec61β1 and SC_Sec61β2, results in heat sensitivity instead of cold sensitivity [22], [24]. Nevertheless, we reasoned that if the function of Sec61β is conserved there should be cross-species complementation in spite of the opposite temperature phenotypes found in *S. pombe* versus *S. cerevisiae*. As shown in Figure 4A, both Sec61β paralogs of *S. cerevisiae* complemented the cold sensitivity of the *S. pombe* Δ*sbh1* strain as efficiently as the endogenous translocon β subunit. Moreover, SC_Sec61β1 and SC_Sec61β2 also fully reversed the SDS sensitivity of the *S. pombe* Δ*sbh1* strain (Figure 4C). Similarly, SP_Sec61β complemented the heat sensitivity of the *sbh1*Δ*sbh2*Δ *S. cerevisiae* strain (Figure 4B). This effect was specific to the translocon β subunit, as the γ subunit homologs of *S. pombe* (SP_Sec61γ) and *S. cerevisiae* (SC_Sec61γ) were unable to complement the temperature-dependent phenotypes in either yeast.

To investigate the possibility of a functional conservation with higher eukaryotes, we transformed the human homolog of Sec61β (HS_Sec61β) in the Sec61β knockout strain of both *S. pombe* and *S. cerevisiae*. Strikingly, HS_Sec61β also complemented the cold sensitivity and SDS sensitivity of the *S. pombe* Δ*sbh1* strain, as well as the heat sensitivity of the *S. cerevisiae sbh1*Δ*sbh2*Δ strain (Figure 4, line 5). Hence, despite distinct phenotypes resulting from the deletion of translocon β subunit in two different yeast species, the function of Sec61β appears to be conserved from yeast to human.

### The transmembrane domain of Sec61β is sufficient for heterologous complementation

**Figure 5:**
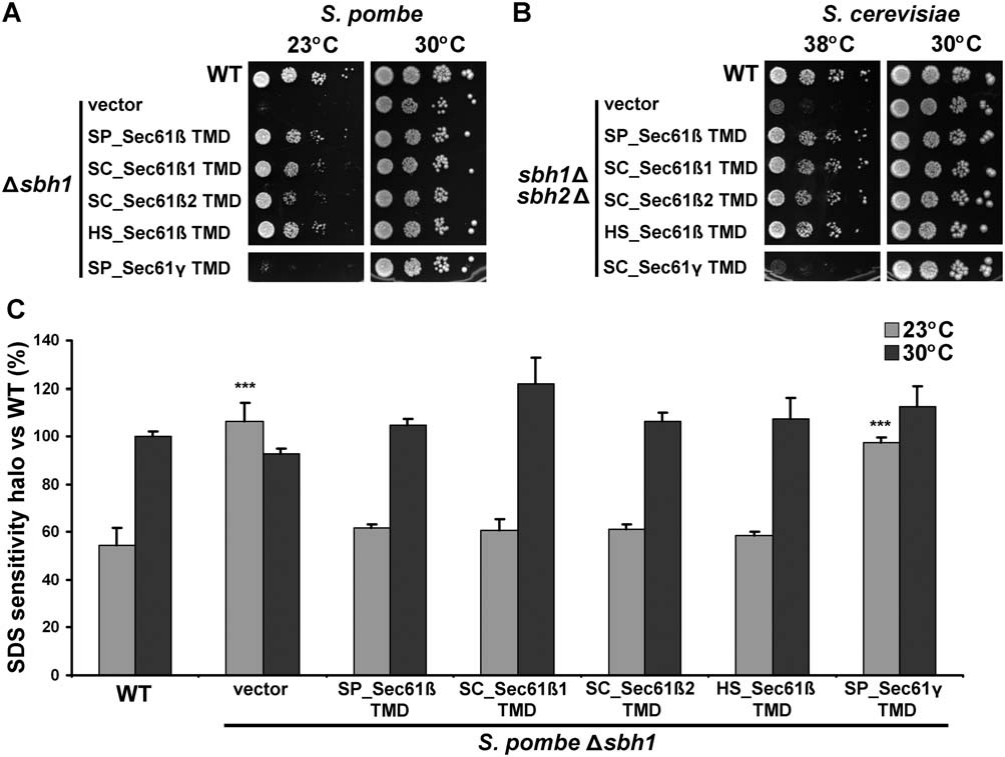
The transmembrane domain (TMD) of different Sec61β homologs is sufficient to complement the knockout of the whole gene in fission and budding yeast. (A) Cold sensitivity of *S. pombe* Δ*sbh1* cells (SP15039) and (B) heat sensitivity of *S. cerevisiae sbh1*Δ*sbh2*Δ cells (SC3232) are rescued by the TMD of different Sec61β homologs. Δ*sbh1* and *sbh1*Δ*sbh2*Δ cells expressing the 26 amino acids of the TMD of Sec61β homologs from *S. pombe* (SP), *S. cerevisiae* (SC) and human (HS) were serial-diluted (10^−1^–10^−4^) and spotted on MM+AL plates or SD-L plates. Growth was monitored during 5 days for *S. cerevisiae* and 7 days for *S. pombe* at the indicated temperatures. Sec61γ from *S. pombe* (SP) or *S. cerevisiae* (SC) were used as negative controls. Results are representative of three or more independent experiments. (C) Suppression of *S. pombe* Δ*sbh1* SDS sensitivity by the TMD of different Sec61β homologs. SDS-sensitivity halo was measured after 3–5 days of incubation at the indicated temperatures for cultures of the strains presented in (A). Data shown are mean±standard deviation of three or more independent experiments. *** indicates *p*<0.001 for Student's t-test versus WT.

Next, we wanted to define the domain of Sec61β responsible for the inter-species complementation. Bioinformatic analyses revealed that the most conserved region of Sec61β in all species studied is mapped to its TMD [47]. Moreover, the TMD of SC_Sec61β1 is sufficient to rescue the heat sensitivity of *sbh1*Δ*sbh2*Δ cells, and to associate with the translocon α and γ subunits [27]. Therefore, we tested if the TMD of different Sec61β homologs could rescue the temperature-sensitive phenotypes in *S. pombe* and *S. cerevisiae* knockout strains. We found that the 26 amino acids of the tail anchor of SP_Sec61β are sufficient to complement the cold sensitivity (Figure 5A), SDS sensitivity (Figure 5C) and secretion defects (Figure 3B) of the *S. pombe* Δ*sbh1* strain to levels equivalent to the full-length protein. The TMDs of the other Sec61β homologs tested were also able to fully complement the defects of the Δ*sbh1* strain, indicating that the fundamental function of the TMD is conserved between species (Figure 5A–C). Likewise, all Sec61β TMDs complemented the heat sensitivity of the *sbh1*Δ*sbh2*Δ *S. cerevisiae* strain (Figure 5B). Again, complementation was specific to the TMD of Sec61β, as the TMD from Sec61γ (Figure 5, line 5) and calnexin (not shown) failed to suppress the phenotypes of the *sbh1*Δ*sbh2*Δ *S. cerevisiae* strain. Collectively, these results indicate that the tail anchor of Sec61β is sufficient to complement the knockout of the entire protein, and that this can be indistinctively accomplished by homologous transmembrane domains ranging from yeast to human. This further confirms that the diverse phenotypes associated with the deletion of Sec61β in distinct species are not due to divergence in the amino acid sequences, but rather they reflect different downstream effects of a conserved Sec61β function supported by the TMD.

## Discussion

We have characterized for the first time the Sec61β homolog from fission yeast. We describe *S. pombe*-specific phenotypes resulting from the overproduction or the lack of this protein, revealing the involvement of SP_Sec61β in cellular morphology, stress resistance and protein secretion. These *S. pombe* phenotypes are distinct from those observed in *S. cerevisiae* [22], [23], [24], [27]. Nevertheless, the Sec61β homologs from *S. pombe*, *S. cerevisiae* and human complement the phenotypes of knockout strains of both fission yeast and budding yeast, to a level comparable to that of the endogenous proteins. Remarkably, the transmembrane domains of Sec61β homologs are sufficient for heterologous complementation, demonstrating that the TMD region supports a fundamental biological function that is conserved across species.

Sec61β is not essential for survival in microorganisms, but is required for development in *Drosophila* [22], [23], [24], [48]. In *S. pombe*, we show that Sec61β is dispensable for growth at optimal temperature, but becomes critical at low temperature (Figure 2–3). Cold sensitivity was also observed for the *Escherichia coli* SecG deletion [21], although it was dependent on a second mutation in *glpR*, involved in glycerol phosphate regulation [49]. In bacteria, the translocation process itself is cold-sensitive, presumably because membrane insertion of the SecA ATPase motor, which is mediated by SecG, is reduced at low temperature [30], [50]. In eukaryotes, a role for Sec61β in cold resistance was proposed in the *Gymnadenia conopsea* psychrophile plant [51]. Thus, the heat-sensitive phenotype observed in *sbh1*Δ*sbh2*Δ cells of *S. cerevisiae* [22], [24] does not reflect a universal phenotype. A possibility is that the differences between the two organisms reflect the prevalence of co- vs post-translational translocation processes in each yeast.

Interestingly, the SDS sensitivity of Δ*sbh1* cells in *S. pombe* contrasts with the SDS resistance of Δ*sbh1* cells observed in *Y. lipolytica* [23]. In addition, in *Y. lipolytica*, cells genetically depleted of Sec61β form colonies displaying morphological changes, while in *S. pombe*, only the overexpression of Sec61β affected cellular morphology and not colony formation. As morphology and SDS resistance are linked to cell-wall biosynthesis, these opposite effects could be due to differences in the composition of the cell-wall of these two yeasts [52]. Since we observed that the translocon β subunit levels also affect cell size, it remains possible that SP_Sec61β is involved in the coordination of cell-membrane growth in fission yeast.

Regarding the effect of Sec61β on secretion, it appears correlated with the efficiency of this process in each yeast. As *S. cerevisiae* is somewhat inefficient for secretion [53], the impact of Sec61β deletion is more apparent in this yeast (47% diminution) [27], while in *Y. lipolytica*, which is more competent for secretion, no difference are observed [23]. In fission yeast, the importance of Sec61β is revealed at 23°C, when the secretion rate of the WT strain is already reduced by 62% as compared to 30°C (Figure 3). The different phenotypes observed for the Sec61β-knockout in *S. pombe* as compared to *S. cerevisiae* and *Y. lipolytica* suggested that different homologs could have distinct functions. However, the lack of the translocon β subunit could affect more severely the secretion of certain proteins, thus providing an alternative explanation for the diverse, and sometimes contrasting, phenotypes associated with deletion or overexpression of Sec61β among species.

Importantly, we show that despite these phenotypic differences, SP_Sec61β is able to complement *sbh1*Δ*sbh2*Δ in budding yeast. Similarly, both SC_Sec61β paralogs complement the cold and SDS sensitivity in *S. pombe*. This is in accordance with previous reports that Sec61β homologs from *Yarrowia lipolytica*, *Kluyveromyces lactis*, *Antonospora locustae* and *Encephalitozoon cuniculi* rescue the heat sensitivity of *S. cerevisiae*, indicating that they are functional homologs [23], [46], [54]. Here we demonstrate that this functional complementation is possible even with higher eukaryotes, as the human homolog of Sec61β also complements the knockout in *S. pombe* and *S. cerevisiae*. These results further suggest that the distinct phenotypes associated with the Sec61β deletion are caused by downstream effects resulting from loss of a common function of the translocon β subunit that can be performed by any homolog.

Phylogenetic studies on Sec61β/Sbh1p/SecG revealed that the consensus region for this protein is located at the hydrophobic peak corresponding to its TMD, while the N-terminal domain is more divergent and disordered [15], [16]. In many archaea, the N-terminal portion of Sec61β homologs is almost twice smaller than their mammalian counterparts. In some eukaryotes, such as parasitic microsporidia, the N-terminal region of Sec61β is practically as short as in archaea, and the protein still complements heat sensitivity of *S. cerevisiae sbh1*Δ*sbh2*Δ cells [54]. Domain mapping of SC_ Sec61β showed that the N-terminal part is dispensable for normal rates of co-translational translocation, heat resistance, association with other translocon subunits and interaction with the SR [27], [28]. This suggests that the TMD of Sec61β is the critical region for protein function, while the larger N-terminal cytosolic part plays secondary roles. In this paper, we show that the fundamental function of the TMD of Sec61β is conserved among distant species. Indeed, the *S. pombe*, *S. cerevisiae* and human TMD homologs complemented various defects caused by Sec61β deletion in fission and budding yeasts, to levels comparable to the entire protein. This property is inherent to the TMD of Sec61β, since other transmembrane sequences did not suppress the phenotypes associated with the Sec61β-knockout in fission and budding yeast.

To date, the only precise function ascribed to the TMD of Sec61β is its role as a tail anchor and ER localization signal. Disruption of this domain results in accumulation of Sec61β in large cytoplasmic puncta [55], [56], while N-terminal fragments of Sec61β devoid of the TMD are unable to complement any known function of the protein in *S. cerevisiae* [27]. Insertion of Sec61β into the ER is not spontaneous, but requires recognition of the TMD by a post-translational targeting machinery, TRC40/Asna1 in mammals or the GET complex in yeast [55], [56]. Thus, it is conceivable that specific residues of the TMD are essential to recruit this complex to the ER membrane. Yet, other tail-anchored proteins, including the essential translocon γ subunit, display lower conservation of the TMD than the β subunit, suggesting a key function for this domain in Sec61β. Indeed, apart from its adaptor role in the translocon, the TMD of SC_Sec61β1 was also found in a distinct complex including Rtn1p, an exocyst-associated protein involved in ER reticulation [57], suggesting additional localizations of Sec61β in the ER [27]. Nevertheless, the TMD of SC_Sec61β2, which is part of the second translocon complex in budding yeast (the Ssh1 complex) [22], [58], [59], also complements cold- and SDS-sensitivity in *S. pombe* (Figure 5). This suggests a redundant and conserved role for this domain. Considering the small size of these hydrophobic sequences, it is unlikely that it exerts a catalytic function, but interactions with conserved transmembrane proteins could mediate the central TMD function. As Sec61β was also shown in crosslinking experiments to form dimers [25], a role of the TMD in translocon oligomerization cannot be excluded [60]. The demonstration of a scaffolding or a recruiting function for the TMD of Sec61β awaits further experimentation.

In sum, our results demonstrate a conserved biological role for the TMD of Sec61β. Our observations support the notion that single-span TMDs have other functions than anchoring in the lipid bilayer. Indeed, many TMDs have been implicated in protein complex assembly, oligomerization, helix packing, transmembrane interactions and signal transduction [61]. Sec61β from *S. pombe* appears as an interesting model for elucidation of the cellular roles of a highly conserved TMD present in every living kingdom.

## Materials and Methods

### Strains and media

*S. pombe* strains were cultured in Edinburgh Minimal Medium (EMM) supplemented with required nutrients [62]. *S. cerevisiae* strains were grown in synthetic defined media (SD) lacking appropriate amino acids [63]. All strains were grown aerobically at 30°C unless stated otherwise. G418 was used at a concentration of 150 µg/mL. Yeast strains used in this study are listed in Table 1.

**Table 1:** Yeast strains used in this study.

Strain	Genotype	Source
*S. pombe*		
SP556	*h− ade6-M216 leu1-32 ura4-D18*	Paul Nurse Lab
SP15039	*h+ ade6-M210 leu1-32 ura4-D18 Δsbh1::kan* ^R^	Bioneer
SP15073	*h+ ade6-M210 his3-D1 leu1-32::adh1p_cel1_3HA_adh1t_kan* ^R^ *ura4-D18*	This study
SP15074	*h− ade6-M216 his3-D1 leu1-32::adh1p_cel1_3HA_adh1t_kan* ^R^ *ura4-D18 Δsbh1::kan* ^R^	This study
*S. cerevisiae*		
NY179	*MATα leu2-3,112 ura3-52*	Peter Novick Lab
SC3232	*MATα sbh1Δ::kan* ^R^ *sbh2Δ::hph* ^R^ *leu2-3,112 ura3-52*	Jussi Jäntti Lab

### Plasmid constructions and transformation

Plasmids used in this study are described in Table 2. All oligonucleotide sequences are available upon request. The coding sequences of *sbh1* ^+^ and *sss1* ^+^ were amplified by PCR from a *S. pombe* cDNA library. Forward primers contained a *Nde*I restriction site followed by a sequence encoding the V5 epitope tag. Reverse primers contained a *Bam*HI restriction site. The entire ORFs were cloned into the pREP42 and pREP2 vectors [42], at the *Nde*I and *Bam*HI sites. pREP42 contains a medium strength thiamine-repressible nmt* promoter, while pREP2 contains a strong *nmt* promoter for overexpression [64]. As previously described in *S. cerevisiae* [27], addition of a N-terminal tag on Sec61β had no effect on overexpression phenotypes nor complementation phenotypes (not shown). Amplification of the *SBH1* and *SBH2* genes was similarly achieved except that *S. cerevisiae* genomic DNA was used and no V5 tag was added. Amplification of *SEC61B* was carried out using a human cDNA bank as template. No V5 tag was added, and a *Bgl*II site was used instead of *Bam*HI. Cloning of *SBH1*, *SBH2* and *SEC61B* in pREP42 was performed as for *sbh1* ^+^. For TMD constructs, the sequences encoding the 26 amino acids of the conserved transmembrane domains of each protein, as mapped by HMMTOP [65], were cloned (see Figure 1 for amino-acid sequences). All TMD sequences were amplified to encode a V5 tag on the N-terminal side of the protein, and inserted into pREP42 using *Nde*I and *Bam*HI restriction sites. For cloning in p415, amplification of *sbh1* ^+^, *SBH1*, *SBH2*, *SEC61B* and TMD constructs was carried out as before but with primers containing *Bam*HI and *Xho*I restriction sites instead of *Nde*I and *Bam*HI, respectively. *SSS1* and *SSS1*_*TMD* were amplified from *S. cerevisiae* genomic DNA, adding a c-MYC tag instead of a V5 tag. The amplified sequences were inserted into the *Bam*HI/*Xho*I restriction sites of p415. All constructs were verified using standard DNA sequencing methods. Transformations were done by the PEG (Polyethylene glycol)/lithium acetate procedure [66]. Plasmids derived from pREP42 were transformed into SP15039 and SP15074 (see below), while plasmids derived from pREP2 were transformed into SP556 and SP15073. Plasmids derived from p415 were transformed into SC3232. Transformations were confirmed by prototrophy and Western blotting.

**Table 2:** Plasmids used in this study.

Plasmid	Features	Source
*S. pombe*		
pREP42	Multicopy *S. pombe* expression vector with *ura4* ^+^ selectable marker and mild nmt* promoter	[42], [70]
pREP42-SP_*sbh1*	pREP42 containing *S. pombe sbh1* ^+^ tagged with V5 (N-terminal)	This study
pREP42-SC_*SBH1*	pREP42 containing *S. cerevisiae SBH1*	This study
pREP42-SC_*SBH2*	pREP42 containing *S. cerevisiae SBH2*	This study
pREP42-HS_*SEC61B*	pREP42 containing human *SEC61B*	This study
pREP42-SP_*sss1*	pREP42 containing *S. pombe sss1* ^+^ tagged with V5 (N-terminal)	This study
pREP42-SP_*sbh1TMD*	pREP42 containing *S. pombe* V5-*sbh1_TMD* (amino acids 68 to 93)	This study
pREP42-SC_ *SBH1TMD*	pREP42 containing *S. cerevisiae* V5*-SBH1_TMD* (amino acids 50 to 75)	This study
pREP42-SC_ *SBH2TMD*	pREP42 containing *S. cerevisiae* V5*-SBH2_TMD* (amino acids 57 to 82)	This study
pREP42-HS_ *SEC61BTMD*	pREP42 containing human V5*-SEC61B_TMD* (amino acids 66 to 91)	This study
pREP42-SP_*sss1TMD*	pREP42 containing *S. pombe* V5*- sss1_TMD* (amino acids 30 to 55)	This study
pREP2	Multicopy *S. pombe* expression vector with *ura4* ^+^ selectable marker and strong *nmt* promoter	[42], [70]
pREP2-SP_*sbh1*	pREP2 containing *S. pombe sbh1* ^+^ tagged with V5 (N-terminal)	This study
pREP2-SP_*sss1*	pREP2 containing *S. pombe sss1* ^+^ tagged with V5 (N-terminal)	This study
*S. cerevisiae*		
p415	pRS415 containing Venus fluorescent protein under *ADH1* promoter and *LEU2* marker. (Venus was removed in the other constructions)	Pascal Chartrand Lab
p415-SP_*sbh1*	p415 containing *S. pombe sbh1* ^+^ tagged with V5 (N-terminal)	This study
p415-SC_*SBH1*	p415 containing *S. cerevisiae SBH1*	This study
p415-SC_*SBH2*	p415 containing *S. cerevisiae SBH2*	This study
p415-HS_*SEC61B*	p415 containing human *SEC61B*	This study
p415-SC_*SSS1*	p415 containing *S. cerevisiae SSS1* tagged with c-MYC (N-terminal)	This study
p415-SP_*sbh1TMD*	p415 containing *S. pombe* V5-*sbh1_TMD* (amino acids 68 to 93)	This study
p415-SC_ *SBH1TMD*	p415 containing *S. cerevisiae* V5*-SBH1_TMD* (amino acids 50 to 75)	This study
p415-SC_ *SBH2TMD*	p415 containing *S. cerevisiae* V5*-SBH2_TMD* (amino acids 57 to 82)	This study
p415-HS_ *SEC61BTMD*	p415 containing human V5*-SEC61B_TMD* (amino acids 66 to 91)	This study
p415-SC_*SSS1TMD*	p415 containing *S. cerevisiae* c-MYC-*SSS1_TMD* (amino acids 49 to 71)	This study

### Sporulation and mating

Strain SP15039 (Δ*sbh1* ^+^) was obtained by sporulation of the heterozygous diploid BG_3365 (*h+/h+ ade6-M210/ade6-M216 leu1-32/leu1-32 ura4-D18/ura4-D18 sbh1* ^+^ */Δsbh1::kan* ^R^) purchased from Bioneer (Daejeon, Korea). After identification of sporulating diploids by iodine staining, tetrads were dissected on EMM plates supplemented with adenine, uracil, leucine and histidine (AULH), using a Nikon Eclipse E400 micromanipulator. Δ*sbh1* cells were identified as G418 resistant, and the knockout was confirmed by PCR, Northern blotting and Southern blotting. Strains SP15073 and SP15074 were obtained by mating SP15039 with SP11033 (*h− his3-D1 leu1-32::adh1p_cel1_3HA_adh1t_kan* ^R^ *ura4-D18 Δcnx1::his3* ^+^ + pREP41*cnx1* ^+^) [45], which expresses *Aspergillus aculeatus* cellulase I under the control of the *adh1p* promoter. After mating on EMM+AULH plates, sporulation was induced in liquid EMM+G418 medium with low amounts of nitrogen. Asci were then subjected to random spore analysis [67]. Clones bearing the *his3-D1* deletion were identified by histidine auxotrophy. Clones expressing the essential calnexin gene at the *cnx1* ^+^ locus instead of the pREP41*cnx1* ^+^ plasmid (leucine marker) were identified by leucine auxotrophy. Clones expressing cellulase were identified by the blue halo created on plates containing 0.1% (w/v) AZCL-HE-Cellulose chromogenic substrate (MegaZyme, North Rocks, N.S.W., Australia). Δ*sbh1* clones (SP15074) were identified by their cold sensitivity at 23°C. The genomic background of each strain was further confirmed by multiple PCR analyses.

### Microscopy imaging

Differential interference contrast (DIC) Nomarski imaging and DAPI-staining microscopy were carried out as previously described [68]. Microscopy analyses were performed with a fluorescence inverted microscope Nikon TE2000U. Images were acquired using a motion-picture camera CCD coolSnapFX M® 12 bit, and treated with UIC Metamorph® software. Flow cytometry analyses (FACS) were done using a FACS Calibur device (Becton Dickinson Biosciences) on 10,000 cells after dilution of exponential cultures to OD_595_ = 0.5 in PBS buffer (136 mM NaCl, 25 mM KCl, 12 mM NaHPO_4_, 18 mM KH_2_PO_4_ pH 7.4).

### Phenotypic assays

The sodium dodecyl sulfate (SDS) sensitivity measurements were carried out as described previously [69]. Briefly, exponentially growing cells were spread on EMM+AL top agar (0.7%), following which 10 µL of 10% SDS was added on a small 3 M paper at the center of each plate. After 3–5 days of incubation at 30°C or 23°C, area of the sensitivity halo was measured and reported on the WT value at 30°C. For temperature sensitivity assays, exponential cultures of each strain were diluted to OD_595_ = 0.5, serially diluted (10^−1^–10^−4^), spotted on solid media (EMM+AL for *S. pombe* or SD-L for *S. cerevisiae*), and incubated at the indicated temperatures (7 days for *S. pombe* or 5 days for *S. cerevisiae*). All results are representative of at least 3 independent experiments.

### Cellulase secretion assays

Cellulase secretion [45] was quantified by activity halo measurements at 30°C or 23°C. An equivalent of 0.2 OD_595_ of exponentially growing cells derived from strains SP15073 (WT) or SP15074 (Δ*sbh1*) were spotted on EMM+ALH supplemented with 0.1% (w/v) AZCL-HE-Cellulose. The area of the blue halo created by secreted cellulase activity was measured each day during 7 days after time zero, which was adjusted for every strain as the moment when the halo exceeds area of the colony (all colonies were of equal size). Each measure is the mean of 3 plates. Secretion rate was calculated for each strain by averaging the slope of independent experiments and divided them by the control strain value (100%) at the corresponding temperature.
